# Ketamine Causes Mitochondrial Dysfunction in Human Induced Pluripotent Stem Cell-Derived Neurons

**DOI:** 10.1371/journal.pone.0128445

**Published:** 2015-05-28

**Authors:** Hiroyuki Ito, Tokujiro Uchida, Koshi Makita

**Affiliations:** Department of Anesthesiology, Tokyo Medical and Dental University, Graduate School of Medical and Dental Sciences, Yushima, Bunkyo-ku, Tokyo, Japan; University of Nebraska-Lincoln, UNITED STATES

## Abstract

**Purpose:**

Ketamine toxicity has been demonstrated in nonhuman mammalian neurons. To study the toxic effect of ketamine on human neurons, an experimental model of cultured neurons from human induced pluripotent stem cells (iPSCs) was examined, and the mechanism of its toxicity was investigated.

**Methods:**

Human iPSC-derived dopaminergic neurons were treated with 0, 20, 100 or 500 μM ketamine for 6 and 24 h. Ketamine toxicity was evaluated by quantification of caspase 3/7 activity, reactive oxygen species (ROS) production, mitochondrial membrane potential, ATP concentration, neurotransmitter reuptake activity and NADH/NAD^+^ ratio. Mitochondrial morphological change was analyzed by transmission electron microscopy and confocal microscopy.

**Results:**

Twenty-four-hour exposure of iPSC-derived neurons to 500 μM ketamine resulted in a 40% increase in caspase 3/7 activity (*P* < 0.01), 14% increase in ROS production (*P* < 0.01), and 81% reduction in mitochondrial membrane potential (*P* < 0.01), compared with untreated cells. Lower concentration of ketamine (100 μM) decreased the ATP level (22%, *P* < 0.01) and increased the NADH/NAD^+^ ratio (46%, *P* < 0.05) without caspase activation. Transmission electron microscopy showed enhanced mitochondrial fission and autophagocytosis at the 100 μM ketamine concentration, which suggests that mitochondrial dysfunction preceded ROS generation and caspase activation.

**Conclusions:**

We established an *in vitro* model for assessing the neurotoxicity of ketamine in iPSC-derived neurons. The present data indicate that the initial mitochondrial dysfunction and autophagy may be related to its inhibitory effect on the mitochondrial electron transport system, which underlies ketamine-induced neural toxicity. Higher ketamine concentration can induce ROS generation and apoptosis in human neurons.

## Introduction

Ketamine is widely used in general anesthesia, perioperative sedation and analgesia. However, recent studies have shown the possibility of neurotoxicity of ketamine in rodents and nonhuman primate neonatal brains [1–6]. These studies have shown that exposure to ketamine during development could result in activation of apoptosis in the early phase of development, and may cause cognitive deficiencies during later developmental stages. Despite the accumulation of data from animal studies regarding the neurotoxicity of ketamine, there remains controversy as to whether these results can be extended to human neonates. Furthermore, the mechanism underlying the neurotoxicity of ketamine has not been fully shown. In this context, there are some advantages in using cell lines established from human tissues as experimental models to study the cellular responses to toxic agents and to overcome interspecies differences and ethical issues. Recently, ketamine-induced neural apoptosis has been demonstrated in human embryonic stem cell (hESC)-derived neurons [7, 8]. These are landmark studies that have shown the mechanism of toxicity of anesthetics in human neurons. However, ethical issues regarding the use of human embryos remain problematic [9–11].

Human induced pluripotent stem cells (iPSC) are generated by epigenetic reprogramming of somatic cells through forced exogenous expression of specific transcription factors [12]. Human iPSCs have characteristics very similar to hESCs, and have the potential to differentiate into the three germ layers of the human body. Furthermore, without the need of embryos for generating human iPSCs, the ethical issues are not as much of a concern. Thus, iPSCs can serve as the basis for the development of drug toxicity tests [13, 14]. Therefore, the establishment of experimental models using human iPSC- (rather than hESC-) derived neurons may lead to easier and more reproducible experiments to study the neurotoxicity of anesthetics in human neurons.

The first objective of this study was to test whether human iPSC-derived neurons could be used as an experimental model for investigating the neurotoxicity of ketamine. For this purpose, we treated cultured human iPSC-derived neurons with various concentrations of ketamine and studied their cellular responses. In the clinical setting, the plasma level of ketamine increases to approximately 100 μM for anesthesia induction, and 15–20 μM ketamine is required for maintaining anesthesia [15–17]. In the *in vitro* cell culture model, a neurotoxic effect has been observed by a wide range of ketamine concentrations (10–3000 μM) after 24 h [7, 8, 18–20]. Thus, we treated the iPSC-derived neurons with increasing doses of ketamine (20, 100, 500 μM) for 6 and 24 h. We also studied the effect of ketamine on a cell line derived from cortical neurons of a 14-week-old human fetal brain. These cells were used to assess the reproducibility of the results obtained from the human iPSC-derived neurons. Upon validation of this experimental model, the second objective was to show the mechanism of ketamine toxicity in human neurons.

## Materials and Methods

### Cell culture

#### (1) Human iPSC-derived neurons

Human dopaminergic neurons were differentiated from cultured human iPSC-derived neural progenitor cells for 14 days using the ReproNeuro DA kit (ReproCELL, Yokohama, Japan). These iPSC-derived neuronal progenitor cells were derived from a single iPSC line, which was established from human somatic cells. The cells were cultured (3 × 10^4^ cells per well) in 96-well plates (Corning Costar Corporation, Corning, NY, USA). Wells were precoated with 0.01% poly-L-lysine (Sigma-Aldrich, St Louis, MO, USA) and Coating Solution (ReproCELL). Cells were cultured for 14 days with Maturation Medium (ReproCELL) at 37°C in a humidified incubator with 95% air and 5% CO_2_. The medium was replaced 3, 7 and 14 days after seeding.

#### (2) Immortalized human neural progenitor cell line

An immortalized human neural progenitor cell line (ReNcell CX; Millipore, Billerica, MA, USA) was used to examine the reproducibility of results obtained from human iPSC-derived neurons. This cell line is derived from the brain cortical region of a 14-week-old human fetus. The cells were cultured according to the manufacturer’s instructions, and all experiments were carried out using cells from passages 5 to 20. For experiments, the cells were cultured in 96-well plates precoated with 20 μg/ml laminin, at a density of 2 × 10^3^ cells per well. Cells were cultured for 2 days in ReNcell NSC Maintenance Medium (Millipore) with 20 ng/mL fibroblast growth factor (Millipore) and 20 ng/mL epidermal growth factor (Millipore), and then cultured for 3 days in ReNcell NSC Maintenance Medium without neuronal differentiation growth factors.

### Immunocytochemical characterization of iPSC-derived neurons

The expression of neuronal marker class III beta-tubulin and dopaminergic cell marker tyrosine hydroxylase (TH) was assessed in iPSC-derived neurons cultured on glass bottom plates (μ-Slide 8 well, Ibidi, Martinsried, Germany) precoated as described above. After 14 days of culturing for differentiation, the cells were fixed with 4% paraformaldehyde for 10 min, permeabilized for 5 min with 0.1% Triton X-100, incubated in blocking buffer [1% bovine serum albumin (KPL, Gaithersburg, MD, USA) in PBS] for 1 h, and stained overnight with primary antibodies against neuron-specific class III beta-tubulin, (1:500; BioLegend Japan, Tokyo, Japan) or TH (1:250; Abcam, Cambridge, UK), followed by incubation with the secondary antibodies Alexa Fluor 488 Chicken anti-mouse IgG (Invitrogen, Carlsbad, CA, USA) or Alexa Fluor 647 anti-rabbit IgG (Invitrogen) for 1 h. Cell nuclei were counterstained with 4′,6-diamidino-2-phenylindole (DAPI, 1:500; Molecular Probes, Carlsbad, CA, USA) for 1 h at room temperature. Images were obtained by a confocal microscope (TCS SP8, Leica microsystems, Tokyo, Japan). The positivity of class III beta-tubulin and TH was measured in three separate cultures of each experimental condition, and five fields in each culture were analyzed. We also examined the positivity of class III beta-tubulin in the cortical neuronal cell line after differentiation as described above.

### Ketamine treatment

After differentiation, the iPSC-derived neurons were treated with increasing doses (20, 100, 500 μM) of ketamine (Daiichi Sankyo, Tokyo, Japan) for 6 and 24 h, to examine whether ketamine neurotoxicity is time- and/or dose-dependent.

### Cell viability analysis

Cell viability analysis was included in ApoTox-Glo Triplex Assay kit (Promega, Madison, WI, USA). Glycyl phenylalanyl-aminofluorocoumarin, a fluorogenic, cell-permeant, peptide substrate was added to assess cell viability after 6 or 24 h of treatment with ketamine following the manufacturer’s protocol. The substrate enters intact cells, where it is cleaved by the live-cell protease activity to generate a fluorescent signal proportional to the number of living cells. Cell viability was assessed by measuring fluorescence with a GloMax Microplate Reader (Promega), using an excitation wavelength of 400 nm and an emission wavelength of 505 nm.

### Caspase 3/7 activity

ApoTox-Glo Triplex Assay (Promega) was used for assessing caspase 3/7 activity in neurons. After the measurement of cell viability described above, the caspase-Glo 3/7 reagent was added into each well, and the plates were briefly mixed by an orbital shaker and incubated for 30 min at 37°C. In this assay, activated caspases cleave a luminogenic peptide substrate, which releases a luminescent signal by a luciferin/luciferase reaction. Caspase activation was determined by measuring luminescence with a GloMAX Microplate Reader.

### Measurement of Reactive Oxygen Species (ROS)

ROS-Glo H_2_O_2_ Assay (Promega) was used to measure changes in the level of ROS by directly detecting H_2_O_2_ in neurons. The cells were plated in white, clear-bottom 96-well tissue culture plates (BD Falcon; BD Biosciences, Franklin Lakes, NJ, USA) for differentiation, and then treated with ketamine-containing media for 6 or 24 h. H_2_O_2_ substrate solution (25 μM) was added to each well, and incubated for 6 h in the presence of ketamine at 37°C in a CO_2_ incubator. H_2_O_2_ substrate reacts directly with H_2_O_2_ in neurons to generate a luciferin precursor. After the incubation with H_2_O_2_, ROS-Glo Detection Solution was added to each well followed by 20 min incubation at 25°C to generate a luminescent signal. Luminescence was measured using a GloMAX Microplate Reader.

To determine if ROS production mediates the activation of caspase 3/7, differentiated neurons were treated with ketamine with or without Trolox, a ROS scavenger. The cells were treated with 500 μM ketamine for 6 h with or without 500 μM Trolox. To examine ROS production in neurons, ROS-Glo H_2_O_2_ Assay was used in the same manner as described above. Additionally, caspase 3/7 activity in neurons was evaluated after treatment with 500 μM ketamine for 6 h with or without 500 μM Trolox, using the caspase-Glo 3/7 reagent as described above.

### Analysis of ATP concentration

Cellular ATP concentration was assessed using the Mitochondrial ToxGlo Assay (Promega). ATP Detection Reagent containing luciferin, ATPase inhibitors and thermostable Ultra-Glo luciferase was added into each well after 6 or 24 h of treatment with ketamine. Cells were lysed and a luminescent signal was generated proportional to the amount of ATP. After mixing the plates with an orbital shaker for 5 min, ATP concentration was determined by measuring luminescence with a GloMAX Microplate Reader.

### Neurotransmitter reuptake assay

The effect of ketamine on monoamine neurotransmitter (dopamine, norepinephrine, serotonin) reuptake activity was examined in iPSC-derived neurons. A Neurotransmitter Transporter Uptake Assay Kit (Molecular Devices, Sunnyvale, CA, USA) was used to measure the neurotransmitter transporter activity, following the manufacturer’s protocol. The kit uses a fluorescent substrate that mimics the biogenic amine neurotransmitters and enters the cell through specific transporters. This results in increased intracellular fluorescence intensity that is monitored in real time using a bottom-reading microplate reader (FLIPR TETRA, Molecular Devices). After neurons were treated with each concentration of ketamine for 24 h, the medium was removed and ketamine in Hank’s balanced salt solution (HBSS, Wako, Osaka, Japan) with 0.1% bovine serum albumin (Sigma-Aldrich) buffer was added to the neuronal cultures. The cultures were then incubated for 30 min at 37°C. Finally, the cells were incubated with Dye Solution for 30 min and were analyzed with a bottom-reading microplate reader in kinetic mode for 30 min using ScreenWorks software version 2.0 (Molecular Devices). As a control, the dopamine reuptake inhibitor, GBR12909 (50 μM, Sigma-Aldrich), was added to each well prior to dispensing Dye Solution.

### NAD^+^ and NADH quantification

To examine the level of oxidative stress induced by ketamine, NAD^+^ (oxidized NAD) and NADH (reduced NAD) concentrations were measured, and the NADH/NAD^+^ ratio was calculated. NAD^+^ and NADH were measured using NAD/NADH-Glo Assay kit (Promega). Briefly, the NAD cycling enzyme is used to convert NAD^+^ to NADH. In the presence of NADH, the enzyme reductase reduces a proluciferin reductase substrate to form luciferin. Then, luciferin is quantified using Ultra-Glo recombinant luciferase, and the light signal produced is proportional to the amount of NAD^+^ and NADH in the neurons. Luminescence was measured with a GloMax Microplate Reader. For a positive control, 10 nM rotenone (Sigma-Aldrich), a mitochondrial complex I inhibitor, was added to each well for 24 h prior to the experiment.

### Evaluation of the mitochondrial respiratory chain and oxidative phosphorylation system

The direct effect of ketamine on the electron transport chain complexes (I, II, IV and V) was measured using the MitoTox Complete OXPHOS Activity Assay Panel (Abcam, Cambridge, MA, USA) following the manufacturer’s protocol. Each of the complexes was obtained from isolated bovine heart mitochondria in their functionally active state using highly specific monoclonal antibodies attached to 96-well microplates. For each of the complexes treated with ketamine (31, 125, 500 μM), complex activity was determined by measuring the decrease in absorbance in milli-optical density per min at room temperature or 37°C, as described in the manufacturer’s protocol. Specified wavelengths (340 nm for complexes I and V, 600 nm for complex II, and 550 nm for complex IV) in kinetic mode (every min for 2 h) were used to measure the absorbance, using a FLUOstar OPTIMA-6 (BMG Labtech, Durham, NC, USA) microplate reader.

### Mitochondrial membrane potential assay

Human iPSC-derived neurons were incubated in black, clear-bottom 96-well tissue culture dishes (BD Falcon; BD Biosciences) for differentiation, and then cultured with ketamine-containing media. After 6 or 24 h of treatment, the mitochondrial membrane potential was determined using Mito-ID Membrane Potential Cytotoxicity Kit (Enzo Life Sciences, Farmingdale, NY, USA). In energized cells, the mitochondria produce an orange fluorescence signal following aggregation of the Mito-ID dye. Mito-ID Membrane Potential Dye Loading Solution was added to each well, followed by 30 min incubation at room temperature. After incubation, the resulting fluorescence was measured with a GloMax Microplate Reader using an excitation wavelength of 480 nm and an emission wavelength of 590 nm. For a positive control, 4 μM carbonyl cyanide 3-chlorophenylhydrazone, an uncoupler of oxidative phosphorylation, was added to each well prior to dispensing Mito-ID Membrane Potential Dye Loading Solution.

### Morphological assessment of active mitochondria by fluorescent imaging

Human iPSC-derived neurons cultured in Poly-L-Lysine-coated multiwell glass bottom dishes (Matsunami, Osaka, Japan) were treated with ketamine-supplemented media for 24 h. Cells were then incubated with a fluorescent probe (MitoTracker Red CMXRos, 100 nM, Invitrogen) for labeling active mitochondria for 30 min. Next, cells were fixed with 4% paraformaldehyde for 10 min, permeabilized for 5 min with 0.1% Triton X-100, incubated with blocking buffer for 1 h and then overnight with primary antibodies against neuron-specific class III beta-tubulin, followed by incubation with the secondary antibodies, Alexa Fluor 488 Chicken anti-mouse IgG. Cell nuclei were counterstained with 4′,6-diamidino-2-phenylindole (DAPI) for 1 h at room temperature. Images were obtained by a confocal microscope (TCS SP8).

### Ultrastructural analysis by electron microscopy

Human iPSC-derived neurons cultured in 96-well tissue culture plates were treated with ketamine-supplemented media for 24 h. The cells were then fixed with 2.5% glutaraldehyde and 4% paraformaldehyde in 0.1 M phosphate-buffered saline at 4°C overnight, and washed with the same buffer. After dehydration through a graded methanol series, the cells were embedded in Epon 812 and polymerized overnight at 60°C. Ultrathin (90 nm) sections were cut with a microtome, double stained with uranyl acetate and lead citrate according to standard procedures [21], and then examined by transmission electron microscopy (H-7100; Hitachi, Ibaraki, Japan).

### Statistical analysis

Data obtained from four independent experiments are reported as mean ± SD. Statistical significance between groups was determined by one-way analysis of variance followed by the Dunnett’s test using Stata/IC software (version 11, Stata Corp, College Station, TX, USA). Statistical significance was defined as *P* < 0.05.

## Results

### Differentiation of human iPSC-derived neural progenitor cells into dopaminergic neurons

**Fig 1:**
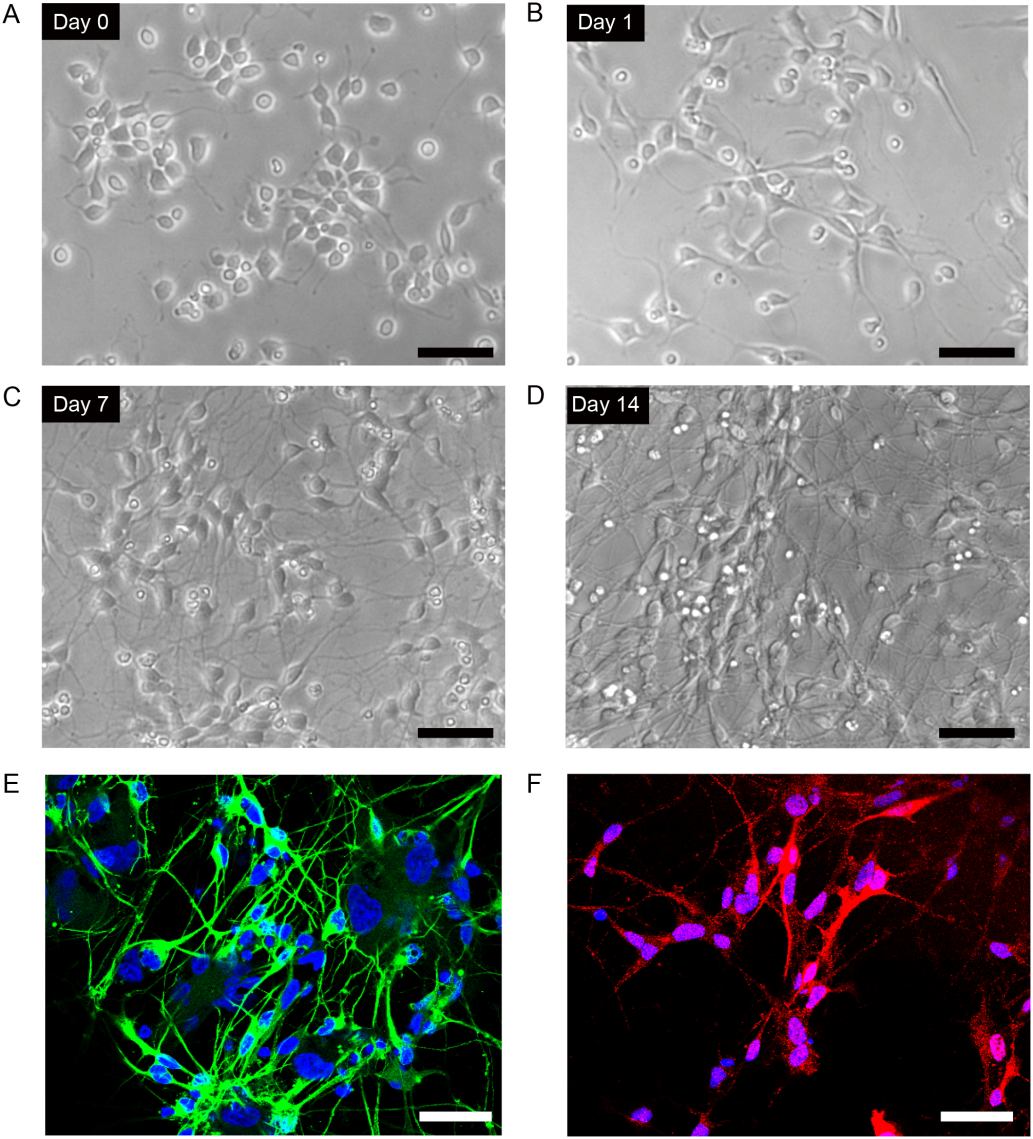
Culture of human iPSC-derived dopaminergic neurons. (A–D) Differentiation of human iPSC-derived neural progenitor cells into dopaminergic neurons from day 0 to 14 after seeding the cells on 96-well plates at 3 × 10^4^ cells per well. (A) day 0 (B) day 1, (C) day 7, (D) day 14. (E) Human dopaminergic neurons (14 days after seeding) stained with anti-beta III-tubulin (green), a neuronal cell marker. (F) Anti tyrosine hydroxylase (red), a dopaminergic neuronal marker. Cells were counterstained with DAPI (blue). Scale bar = 50 μm.

Representative phase contrast images of cultured iPSC-derived dopaminergic neurons are shown in Fig 1. Initially the neural progenitor cells had triangular morphology (Fig 1A and 1B), and then became round with small neuronal processes (Fig 1C). Neuronal processes extended further and extensive networks were formed after 14 days in culture (Fig 1D). The positivity of beta III-tubulin and TH was 91.8 ± 2.9% (Fig 1E) and 36.9 ± 9.7% (Fig 1F), respectively. In the differentiated cortical neuronal cell line, the positive rate of beta III-tubulin was 98.2 ± 1.1%.

### Ketamine induces morphological changes in iPSC-derived neurons

**Fig 2:**
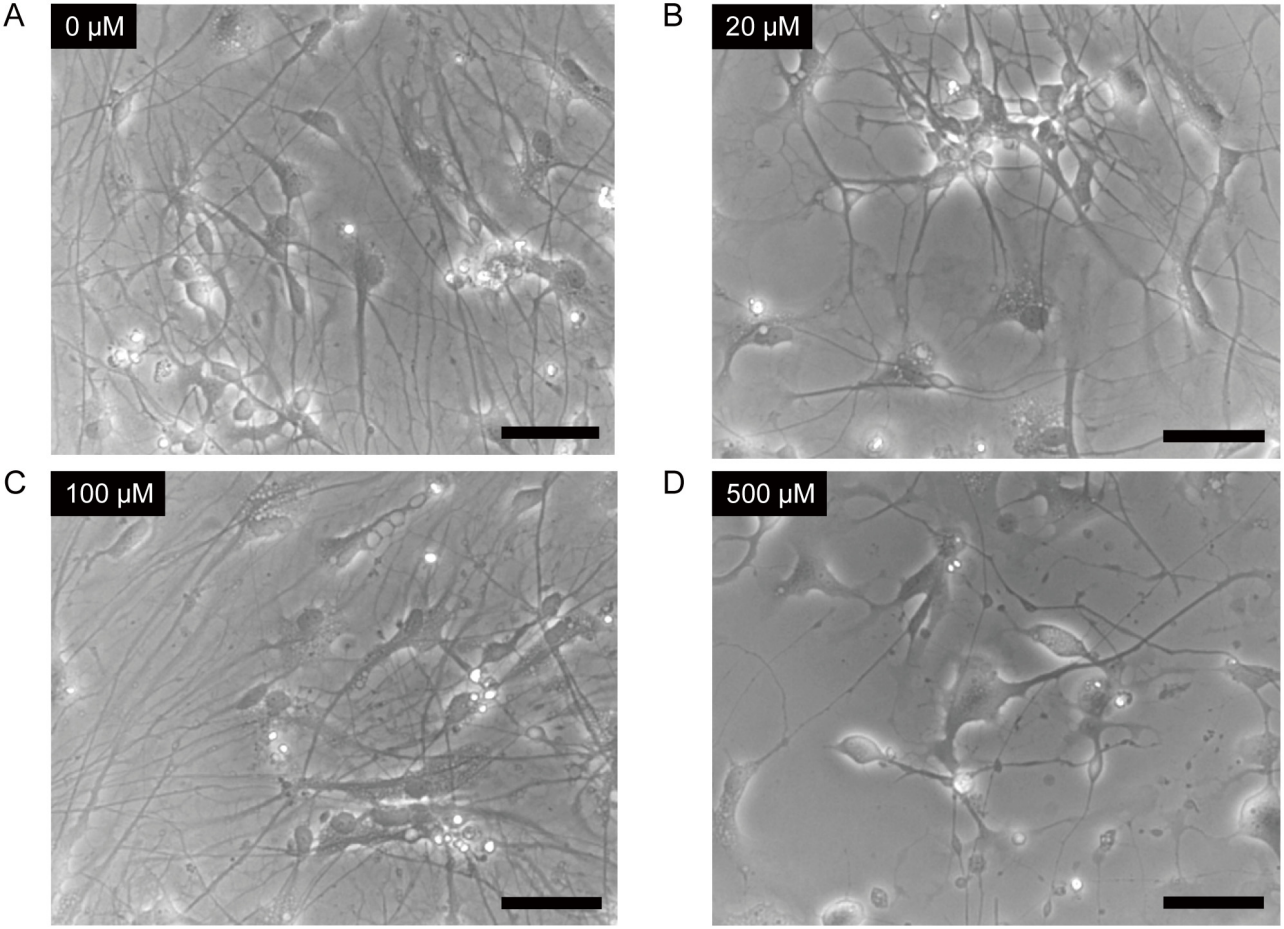
Ketamine induced morphological changes in neurons derived from iPSCs. Cells were treated with 0 μM (A), 20 μM (B), 100 μM (C) or 500 μM (D) ketamine for 24 h. Lower doses (20, 100 μM) of ketamine treatment did not affect the overall cell morphology (B and C). However, 500 μM ketamine caused neuronal processes to retract and it diminished neuronal networks (D). Scale bar = 50 μm.

We examined whether ketamine induces morphological changes in differentiated neurons derived from human iPSCs. iPSC-derived dopaminergic neurons were treated with increasing concentrations of ketamine (20, 100, 500 μM) for 24 h (Fig 2). The lower doses of ketamine (20, 100 μM) did not affect the overall cellular morphology (Fig 2B and 2C). However, 500 μM ketamine caused neuronal process retraction and diminished neuronal networks (Fig 2D).

### Ketamine induces apoptosis and ROS production in iPSC-derived neurons

**Fig 3:**
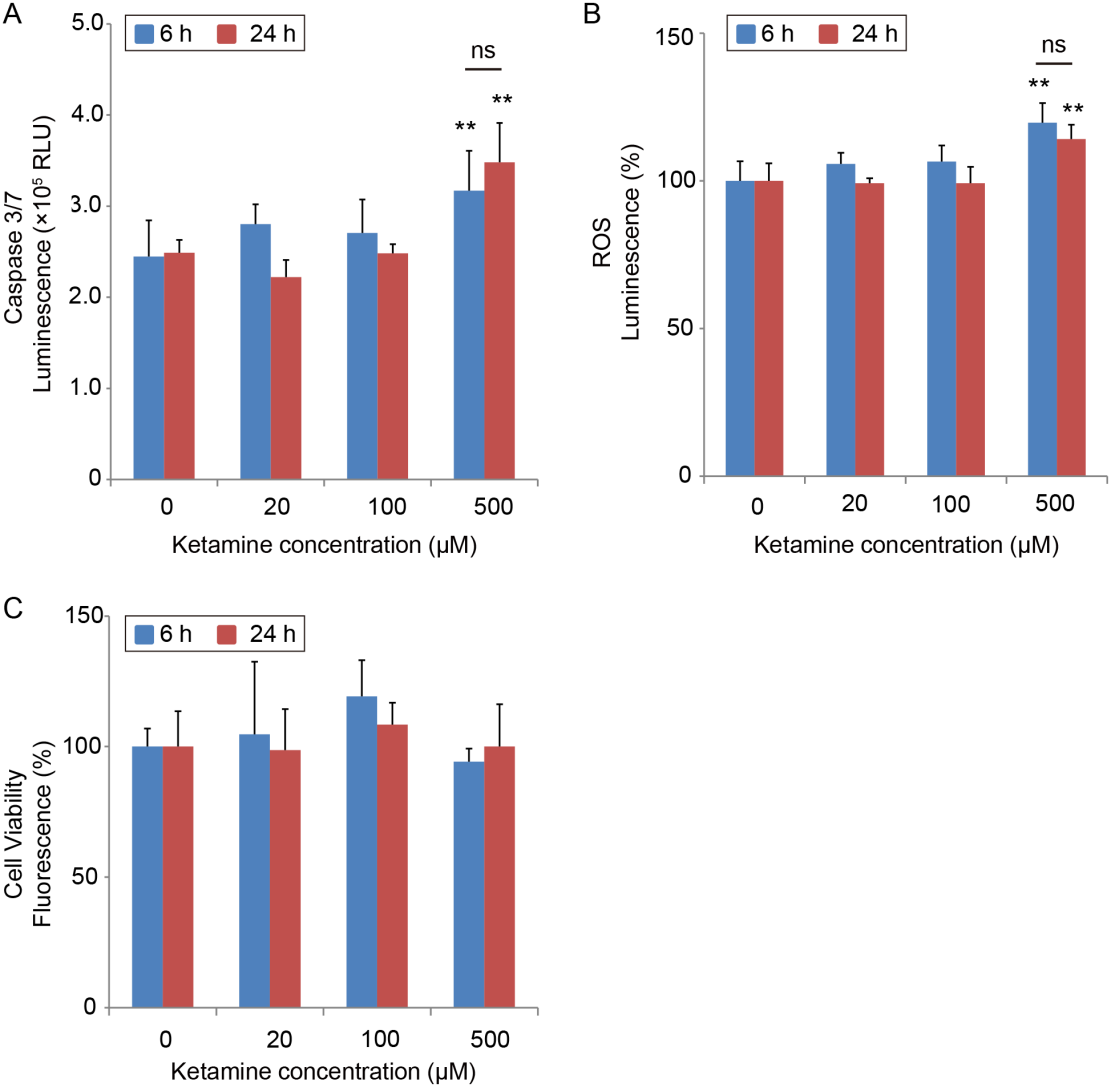
Effect of Ketamine on caspase 3/7 activity and ROS production, and cell viability in cultured iPSC-derived neurons. Neurons were exposed to increasing concentrations (20, 100 and 500 μM) of ketamine for 6 and 24 h. (A) Caspase 3/7 activity was used to evaluate ketamine-induced apoptosis in iPSC-derived neurons. Ketamine (500 μM) increased caspase 3/7 activity after 6 and 24 h of treatment. ROS production was used to evaluate ketamine-induced oxidative stress in iPSC-derived neurons. (B) Ketamine (500 μM) increased ROS production both after 6 and 24 h. (C) Cell viability did not change among all groups. Data are presented as mean ± SD; n = 4 in each experimental condition. ** *P* < 0.01, compared with untreated controls. RLU = relative light units.

Next, we measured caspase 3/7 activity, to determine whether the ketamine-induced neurotoxicity in iPSC-derived neurons was due to apoptosis. Caspase 3/7 activation occurs in the early stages of apoptosis [22]. Fig 3A shows that compared with untreated control cells, treatment with 500 μM ketamine for 6 h significantly increased caspase 3/7 activity, which lasted for 24 h, as it was also observed after the 24-h exposure. Moreover, ROS production was significantly elevated (Fig 3B) following a 6-h treatment with 500 μM ketamine. This elevation was also observed after 24 h of exposure to ketamine. The lower concentrations of ketamine (20, 100 μM) did not activate caspase 3/7 or increase ROS production. Similar results were obtained with the cortical neuronal cell line derived from human fetal brain tissue. After exposure to 500 μM ketamine for 24 h, caspase 3/7 activity and ROS production significantly increased in the cortical neuronal cell line [S1A and S1B Fig]. Furthermore, we assessed cell viability in ketamine-treated iPSC-derived neurons by measuring living cell protease activity. The cell viability did not significantly change upon ketamine treatment for 6 or 24 h (Fig 3C).

**Fig 4:**
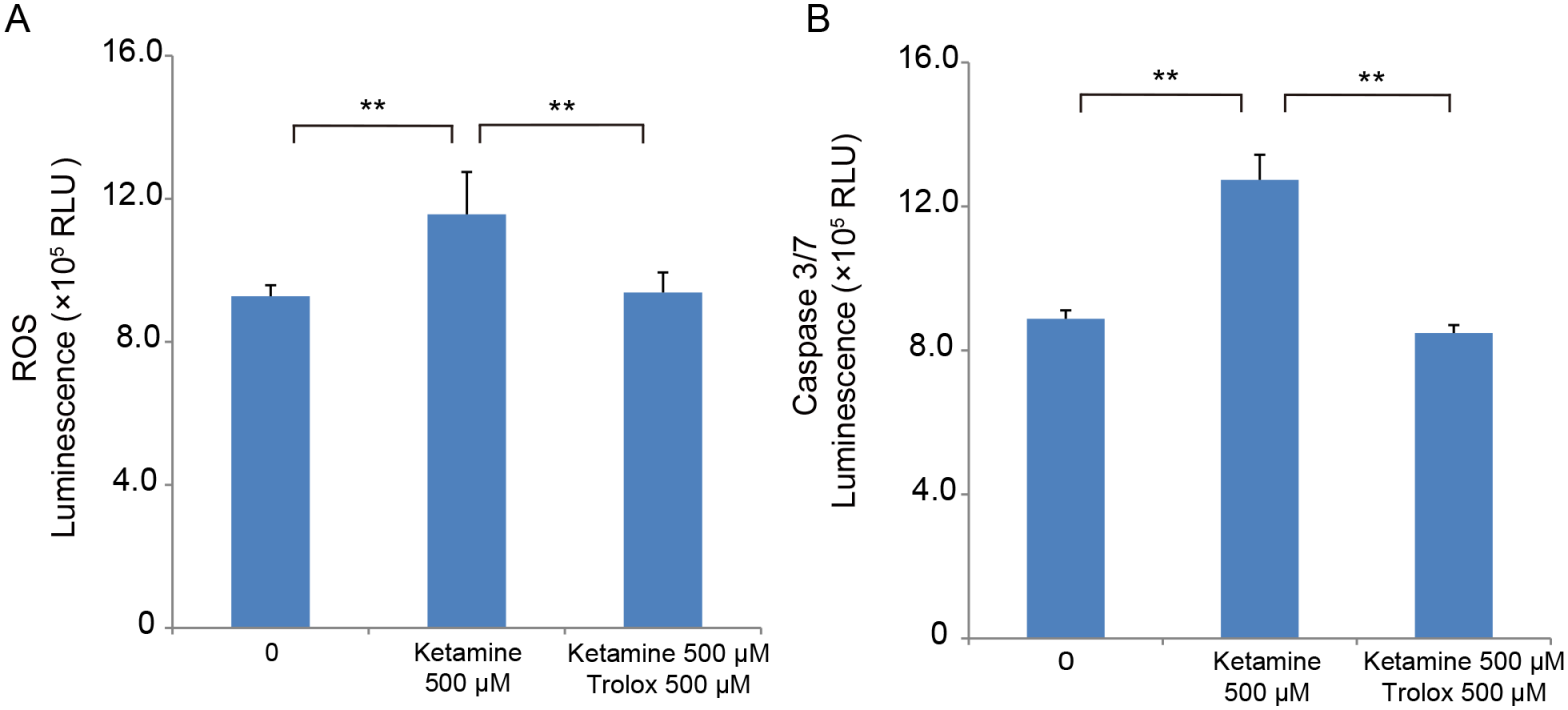
ROS scavenger Trolox attenuates ROS production and caspase 3/7 activation in ketamine-treated neurons. To determine whether ROS production mediates activation of caspase 3/7, iPSC-derived dopaminergic neurons were treated for 6 h with ketamine with or without the ROS scavenger, Trolox. (A) Trolox (500 μM) significantly inhibited ROS generation in the 500 μM ketamine-treated neurons (1.24-fold ± 0.14 ketamine alone vs. 1.01-fold ± 0.06 ketamine with Trolox). (B) Trolox also inhibited caspase 3/7 activation in the 500 μM ketamine-treated neurons (1.43-fold ± 0.12 ketamine alone vs. 1.05-fold ± 0.19 ketamine with Trolox). Results are presented as mean ± SD; n = 4 for each experiment. ** *P* < 0.01, compared with untreated controls.

To study the contribution of ROS production to the activation of caspase 3/7, differentiated iPSC-derived dopaminergic neurons were treated for 6 h with 500 μM ketamine with or without the ROS scavenger, Trolox (Sigma-Aldrich). Trolox (500 μM) significantly inhibited ROS generation in the ketamine-treated neurons (Fig 4A). This inhibition was associated with an attenuation in ketamine-induced caspase 3/7 activation (Fig 4B).

### Ketamine induces mitochondrial dysfunction

**Fig 5:**
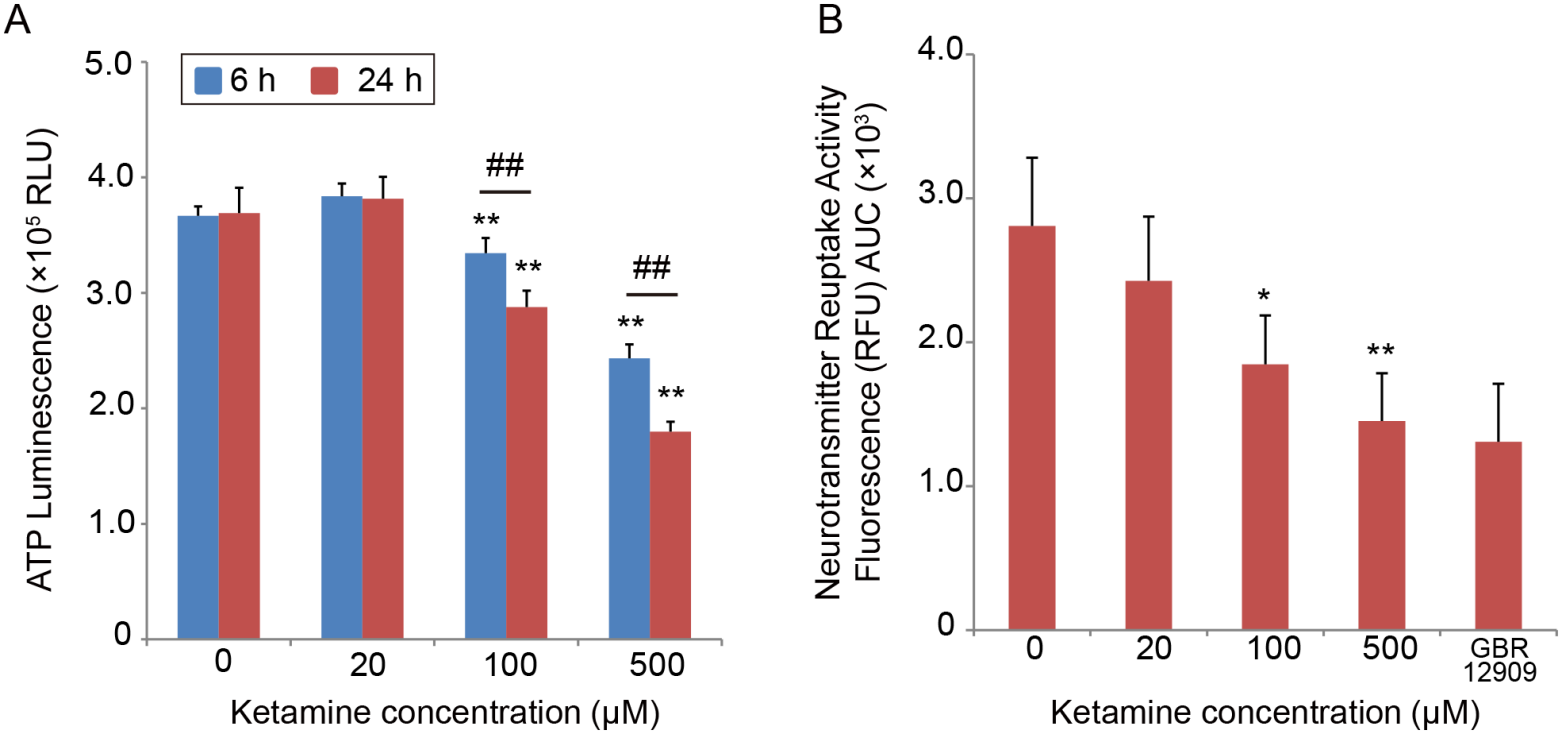
Ketamine decreases the ATP level and neurotransmitter reuptake activity in cultured iPSC-derived neurons. (A) Ketamine decreased the ATP level in a time- and dose-dependent manner. In the 6-h treatment, the ATP level significantly decreased to 91% with 100 μM ketamine, and to 66% with 500 μM ketamine, compared with untreated controls. In the 24-h treatment, the ATP level significantly decreased to 78% with 100 μM ketamine, and to 48% with 500 μM ketamine. (B) Treatment with 100 or 500 μM ketamine for 24 h resulted in decreased neurotransmitter reuptake activity to approximately 65% and 51%, respectively, compared with control cells. Results are presented as mean ± SD; n = 4 for each experiment. * *P* < 0.05, ** *P* < 0.01, compared with untreated controls. ^##^ *P* < 0.01, between the groups. GBR12909 = 1-(2-[bis(4-fluorophenyl)-[methoxy]ethyl)-4-(3-phenylpropyl) piperazine; RLU = relative light units; RFU = relative fluorescence units; AUC = area under the curve.

Next, we studied whether mitochondrial dysfunction is associated with ketamine toxicity. To this end, we measured the ATP level in iPSC-derived neurons. Ketamine significantly decreased the ATP level at 100 and 500 μM after 6 and 24 h of treatment, compared with the control cells (Fig 5A). Compared with the 6-h treatment, treatment for 24 h with 100 or 500 μM ketamine significantly decreased the ATP level. The cortical neuronal cells also showed a significant reduction in the ATP level after treatment with 100 μM or 500 μM ketamine for 24 h [S1C Fig].

We next investigated whether ketamine treatment affects monoamine neurotransmitter reuptake activity in iPSC-derived neurons. Cells treated with 100 or 500 μM ketamine for 24 h showed significantly decreased neurotransmitter reuptake activity, compared with control cells (Fig 5B).

**Fig 6:**
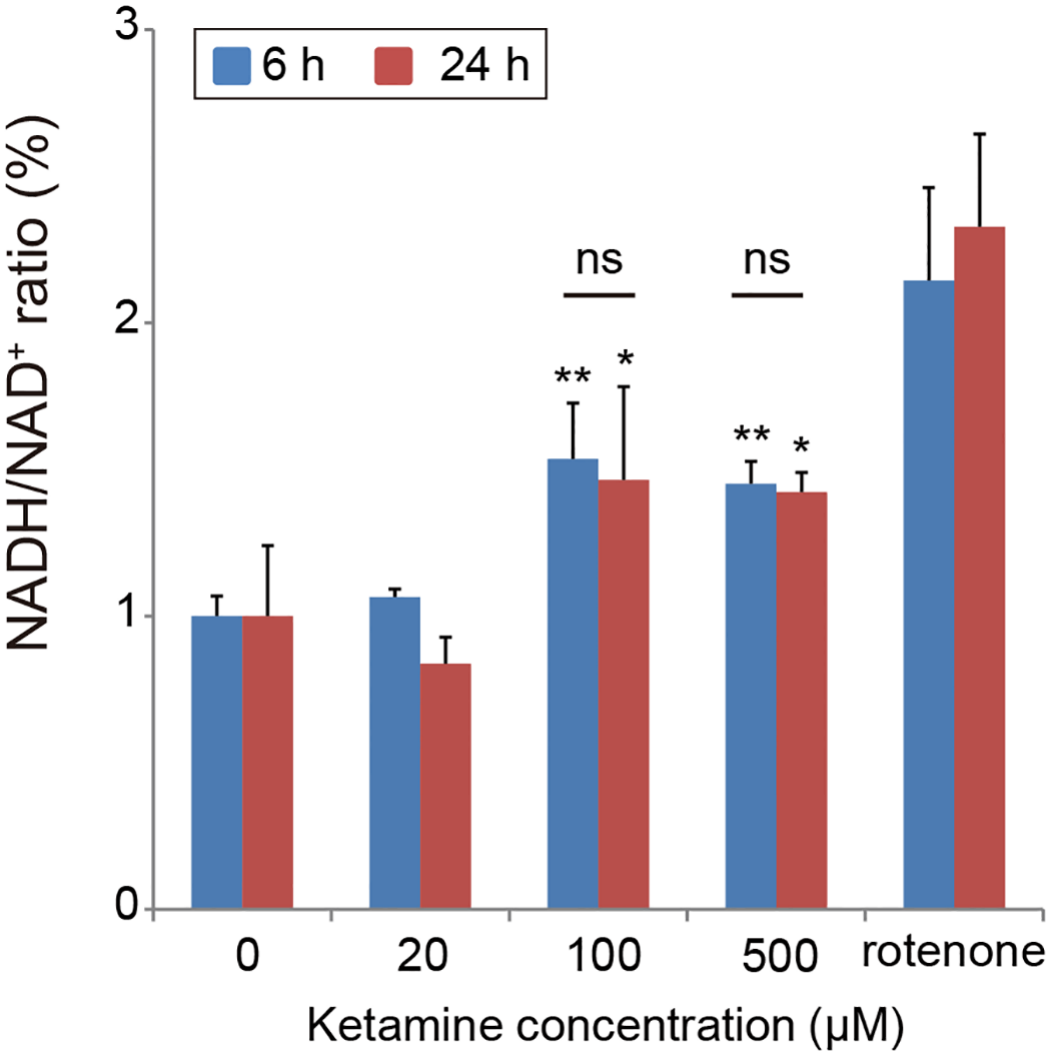
Effect of ketamine on NADH/NAD^+^ ratio in cultured neurons derived from iPSCs. The neurons treated with 100 or 500 μM ketamine showed a significant increase in the NADH/NAD^+^ ratio compared with the control cells both after 6 and 24 h treatment. As a positive control, 10 nM rotenone (24 h) was used. Results are presented as mean ± SD; n = 4 for each experiment. * *P* < 0.05, ** *P* < 0.01, compared with untreated controls. RLU = relative light units.

To examine the effect of ketamine on oxidative stress, we measured the levels of the oxidized and reduced forms of NAD in the iPSC-derived neurons. In both the 6-h and 24-h ketamine treatment experiments, the results showed that ketamine treatment at 100 and 500 μM significantly increased the NADH/NAD^+^ ratio (Fig 6). Moreover, in the cortical neuronal cells, the NADH/NAD^+^ ratio was significantly higher in the 100 and 500 μM ketamine-treated cultures compared with the control group (S2 Fig).

**Fig 7:**
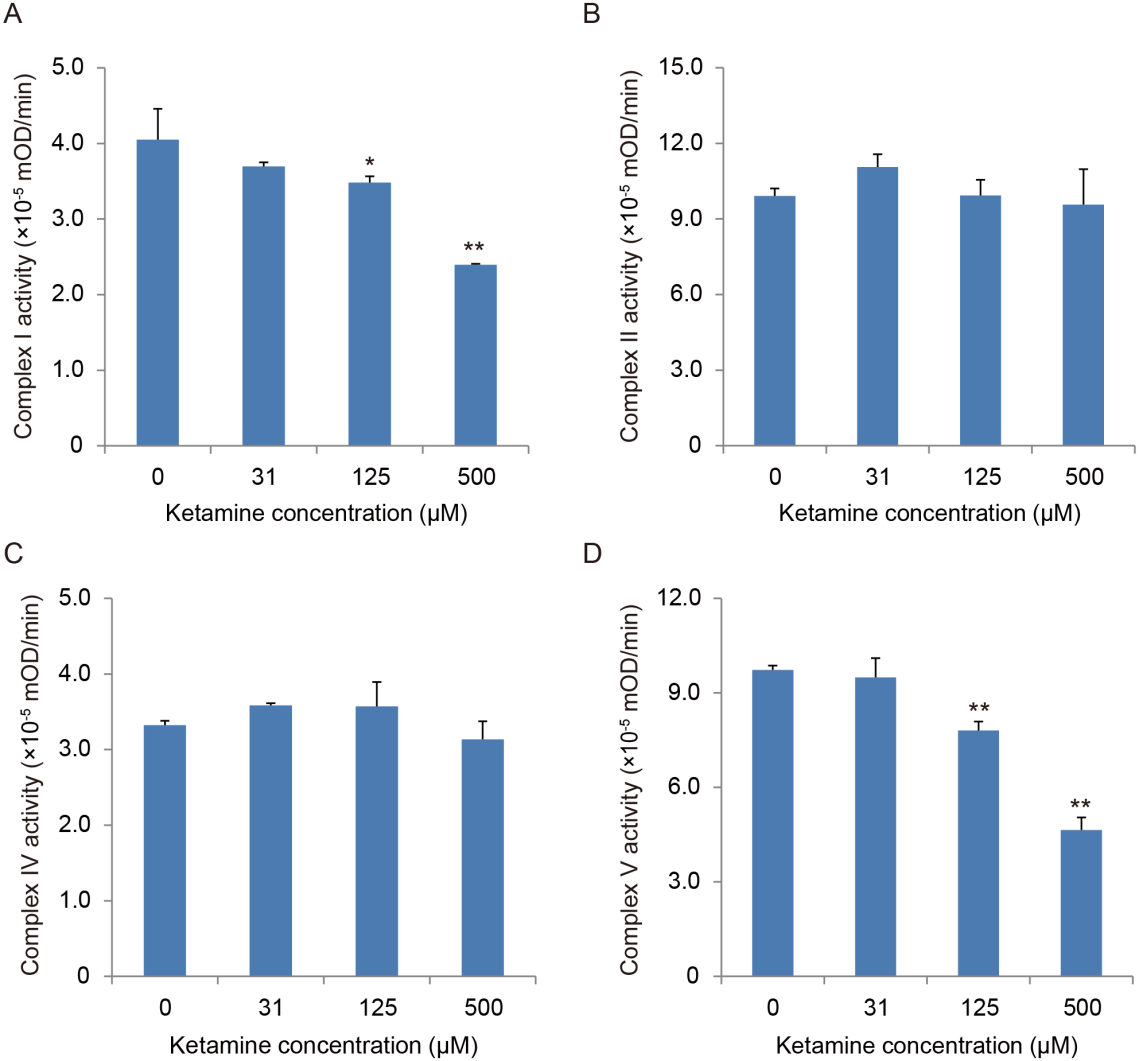
Effect of ketamine on mitochondrial respiratory complexes from bovine heart mitochondria. (A) Complex I (NADH dehydrogenase) assay. (B) Complex II (succinate dehydrogenase) assay. (C) Complex IV (cytochrome c oxidase) assay. (D) Complex V (ATP synthase) assay. Ketamine (≥ 125 μM) significantly reduced the activity of complexes I (A) and complex V (D). Results are presented as mean ± SD; n = 3 in each experiment. * *P* < 0.05, ** *P* < 0.01, compared with control cells.

Based on the finding that 100 and 500 μM ketamine increased the NADH/NAD^+^ ratio in the iPSC-derived neurons, we hypothesized that ketamine can induce dysfunction in the oxidative phosphorylation system in mitochondria, resulting in a decrease in the oxidation of NADH to NAD^+^. To study this hypothesis, we investigated whether ketamine directly affects the activity of mitochondrial respiratory complexes using a biochemical assay in which NADH is oxidized to NAD^+^. The effect of ketamine treatment on bovine heart mitochondrial respiratory chain complex (I, II, IV and V) activities is shown in Fig 7. The activity of Complex I (NADH dehydrogenase; Fig 7A) and complex V (ATP synthase; Fig 7D) significantly decreased after treatment with 125 or 500 μM ketamine. However, complex II (succinate dehydrogenase) and complex IV (cytochrome c oxidase) activities were unchanged even with the highest concentration (500 μM) of ketamine (Fig 7B and 7C, respectively).

**Fig 8:**
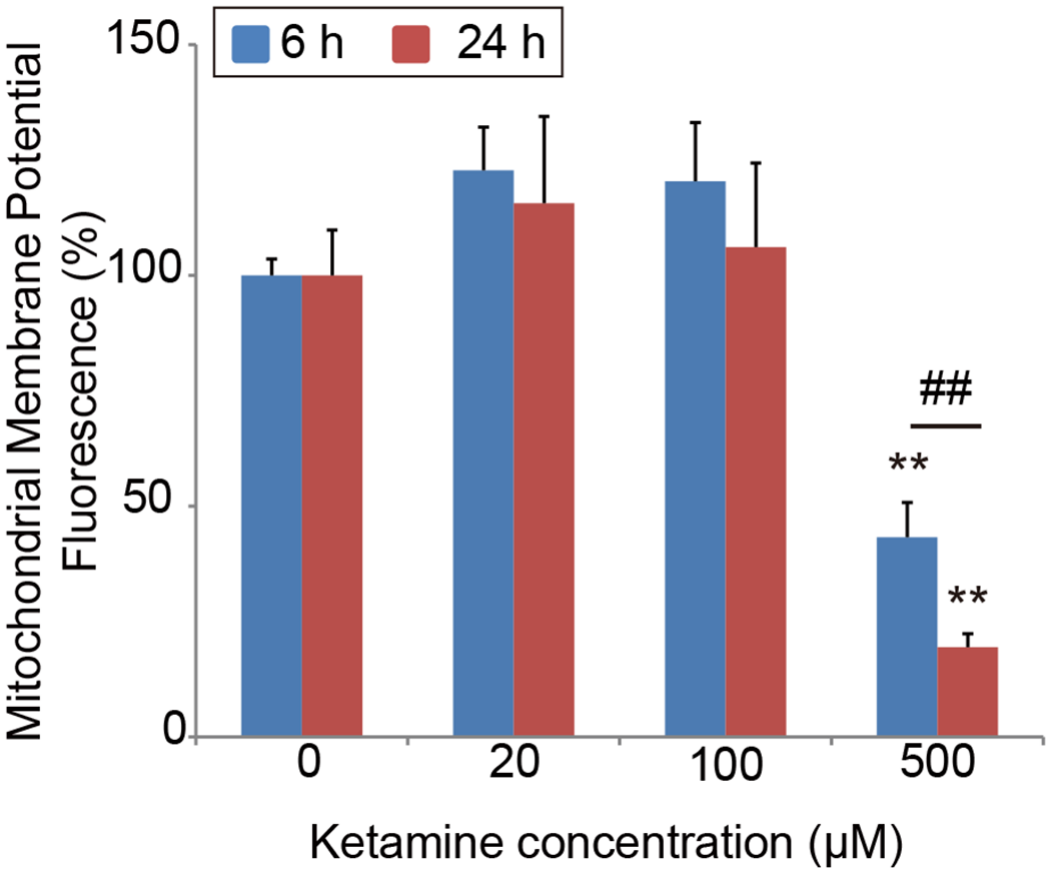
Effect of ketamine on mitochondrial membrane potential in cultured neurons derived from iPSCs. Quantification of mitochondrial membrane potential in ketamine-treated neurons. The cells treated with 500 μM ketamine for 6 and 24 h showed significant reduction in mitochondrial membrane potential. Carbonyl cyanide 3-chlorophenylhydrazone, which disrupts the mitochondrial membrane potential, was used as a positive control. All data were extracted from the fluorescence of 4 μM carbonyl cyanide 3-chlorophenylhydrazone-treated neurons. Results are presented as mean ± SD; n = 4 for each experimental condition. ** *P* < 0.01 compared with untreated controls. ^##^ *P* < 0.01, between the groups.

**Fig 9:**
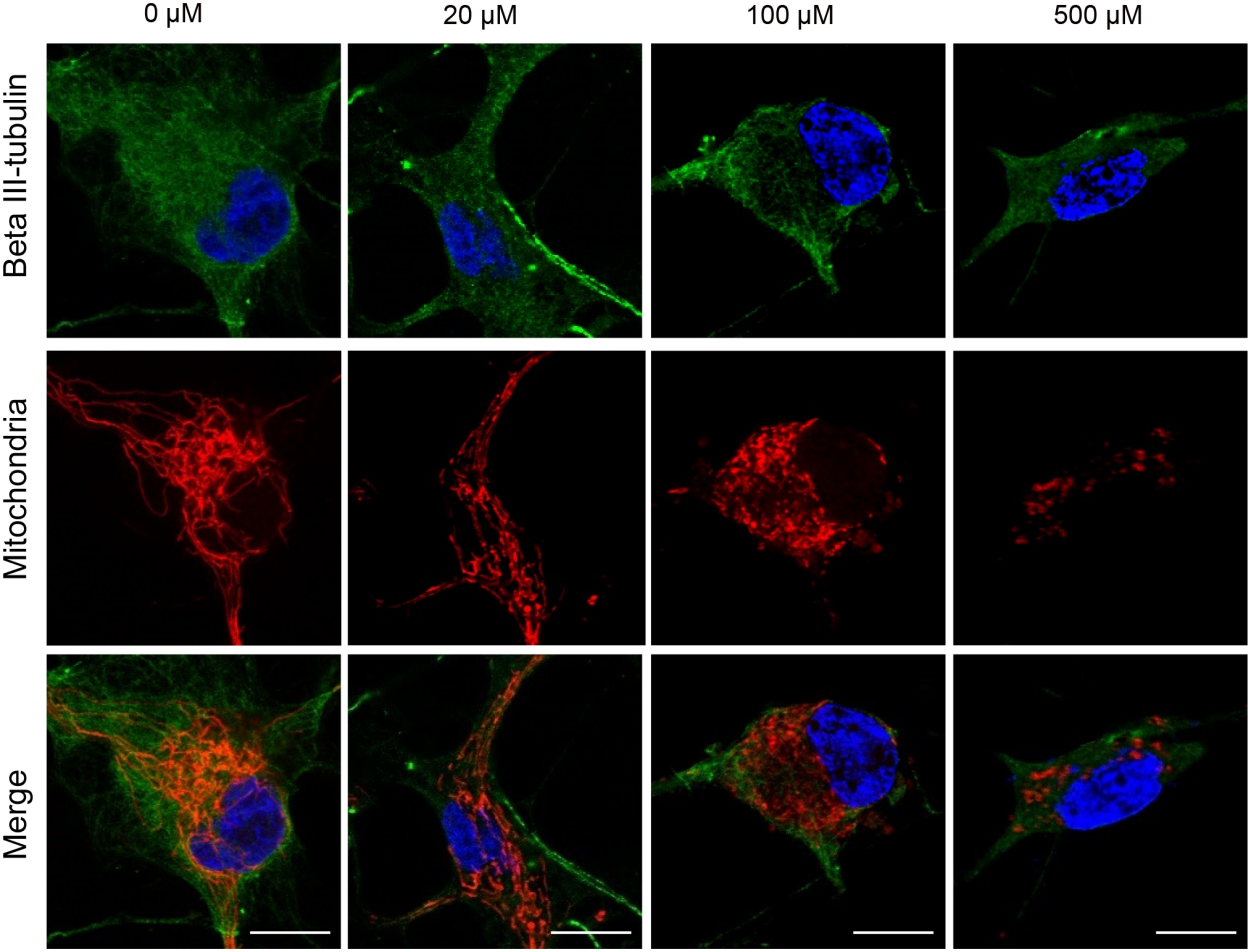
Mitochondrial morphology in iPSC-derived neurons at baseline (Control) and ketamine treatment conditions using confocal microscopy. Cells were treated with ketamine for 24 h, and stained with anti-beta III-tubulin (green), MitoTracker Red CMXRos (red) and DAPI (blue). Cells treated with ketamine at 0 or 20 μM showed elongated mitochondria (red), whereas higher concentrations (100 and 500 μM) of ketamine resulted in small, punctuate mitochondria. The fluorescence intensity of MitoTracker Red was weakened in the 500 μM ketamine-treated neuron. Scale bar = 10 μm.

**Fig 10:**
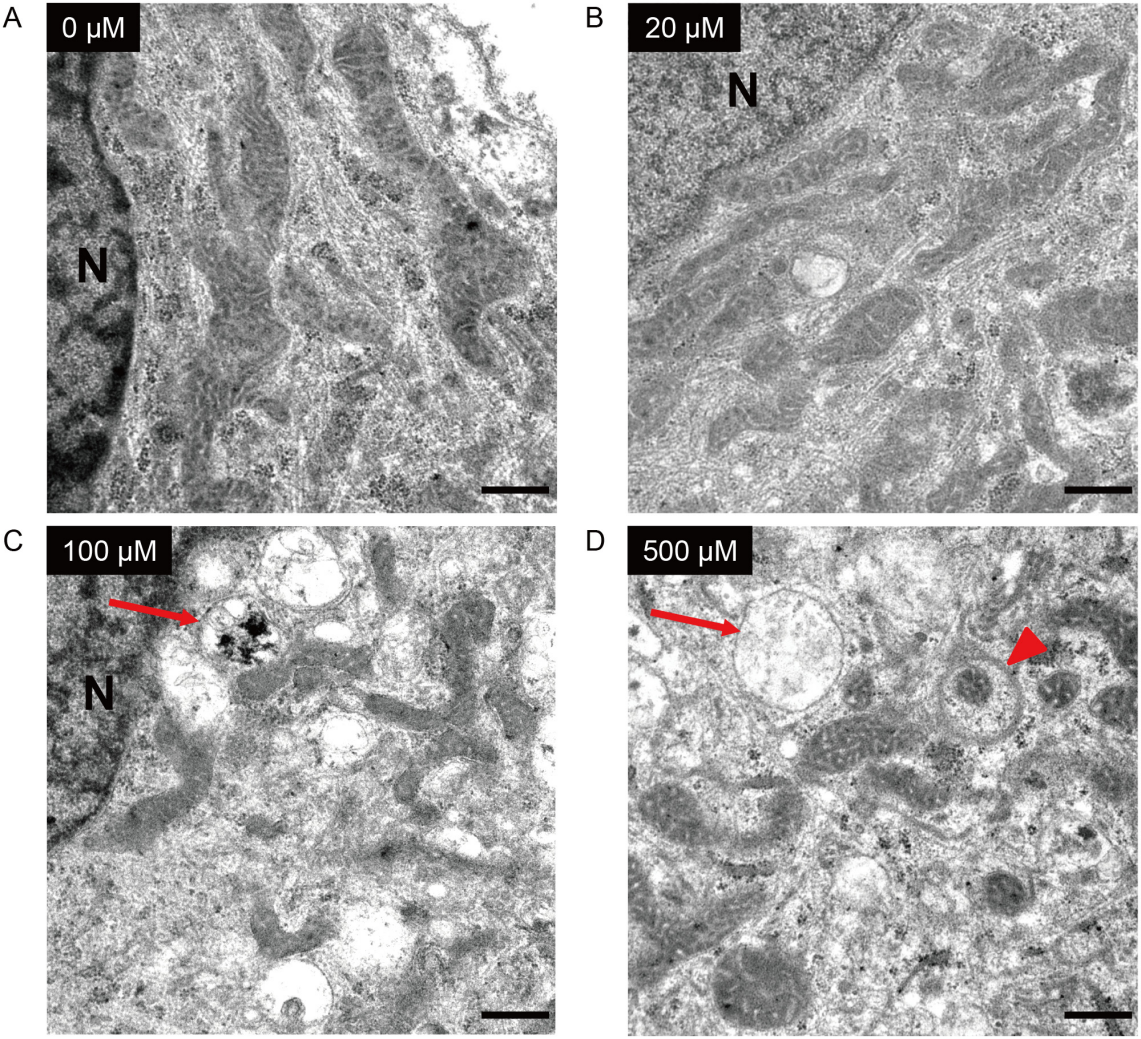
Effect of ketamine on mitochondrial morphology in neurons derived from iPSCs. Cells were treated with ketamine for 24 h, and observed by transmission electron microscopy. Untreated cells (A) and cells treated with 20 μM ketamine (B) had elongated mitochondria with intact inner and outer membranes. Treatment with 100 μM ketamine (C) resulted in fragmented mitochondria and the presence of autophagosomes (arrow). After treatment with 500 μM ketamine (D), the structure of the mitochondria became discrete and round, and the mitochondrial length was shortened. Autophagosomes (arrow) were detected, and some fragmented mitochondria were degraded by autophagosomes (arrowhead). N: nucleus. Scale bar = 500 nm.

We then examined the mitochondrial membrane potential after ketamine treatment. Ketamine treatment at 500 μM for 6 or 24 h significantly decreased the mitochondrial membrane potential compared with the control cells, in both the iPSC-derived neurons (Fig 8) and cortical neuronal cells [S1D Fig]. Next, we investigated the morphological change in activated mitochondria in ketamine-treated neurons. Using confocal microscopy we observed that neurons in the control and 20 μM ketamine treatment for 24 h had elongated mitochondria both in the cell body and in the neurite-like structures (Fig 9). In contrast, neurons in the 100 and 500 μM ketamine treatments had punctate and shortened mitochondria localized in the cell body (Fig 9). Furthermore, we detected ketamine-induced ultrastructural changes in neurons by transmission electron microscope. Fig 10 shows representative images of iPSC-derived neurons treated with ketamine (0, 20, 100, 500 μM) for 24 h. Neurons in the control and 20 μM ketamine-treated cultures had notably elongated mitochondria of varying lengths, some exceeding 4 μm. However, higher concentrations of ketamine caused abnormal ultrastructural changes in the neurons. In the 100 μM ketamine-treated neurons, fragmented mitochondria were observed and autophagosomes were found in the cytosol. In the 500 μM ketamine-treated culture, the lengths of mitochondria were shortened (0.5–1.5 μm long), and the cristae appeared somewhat irregular and disrupted. Moreover, autophagosomes were pronounced and found in almost every cell, often occupying most of the cytosolic volume. We obtained similar findings in the cortical neuronal cells treated with ketamine (S3 Fig).

## Discussion

In this study, we examined the toxic effects of ketamine on cultured human iPSC-derived dopaminergic neurons. The results showed that ketamine is toxic to human iPSC-derived neurons, similarly to its effect on hESCs, as described in previous reports [7, 8]. A high dose of ketamine (500 μM) increased ROS production and activated caspase 3/7 activity, resulting in the dissipation of the mitochondrial membrane potential. Even at the lower concentration, which corresponds to the upper range of the clinical plasma concentration (100 μM), ketamine caused a reduction in the ATP level and neurotransmitter reuptake activity. Both confocal and transmission electron microscopy analyses showed mitochondrial fission at the 100 μM ketamine concentration. Furthermore, mitochondrial dysfunction, which was observed at 100 μM, was accompanied by an increase in the NADH/NAD^+^ ratio, suggesting insufficient consumption of NADH during oxidative phosphorylation. In the biochemical assay for evaluating the direct effect of ketamine on the bovine heart mitochondrial electron transport system, ketamine impaired the activities of complexes I and V.

### Human iPSC-derived neurons as an experimental model for studying ketamine toxicity

Several studies have reported that ketamine is neurotoxic for developing rodents and nonhuman primates [1–3, 23], and that the neurotoxicity occurs in a dose- and time-dependent manner. However, because of the interspecies differences in central nervous system development, neural plasticity and sensitivity to anesthetics, it is not possible to extrapolate these findings directly to the human nervous system. Some human epidemiological studies have demonstrated that children exposed to anesthesia early in life have a higher incidence of learning disabilities later in life [24, 25]. However, it is difficult to exclude the possible effects of confounding factors related to surgery and coexisting diseases. Therefore, the neurotoxicity of anesthetics to the developing human brain is not fully understood. The use of *in vitro* models may be one way to assess the toxicity of various anesthetics to human neurons. In this context, it has recently been reported that ketamine induced apoptosis in neurons derived from hESCs [7, 8]. Although hESCs are useful for testing drug toxicity, their use is restricted because of limited supply and ethical concerns [9–11, 14]. To overcome this limitation, in the current study we used human iPSC-derived neurons.

Treatment of iPSC-derived neurons with a high dose of ketamine (500 μM) resulted in the activation of apoptosis, ROS generation and disruption of the mitochondrial membrane potential. Furthermore, the results obtained from the cortical neuronal cell line were consistent with those from the iPSC-derived neurons. We observed elevated caspase 3/7 levels even after 6 h of treatment with ketamine, which was prevented by the ROS scavenger, Trolox. In the hESC model, Bosnjak *et al*. have reported that apoptosis occurred in neurons treated with 3000 μM ketamine for 24 h [7], and Bai *et al*. have shown that treatment with 100 μM ketamine for 24 h induced neural apoptosis via destruction of mitochondria by ROS [8]. Although there is discrepancy in the toxic range and exposure time of ketamine, this difference may be due to differences in the methods used for cell differentiation and culture media. However, the iPSC-derived neurons had qualitatively the same reactions as the hESC-derived neurons (e.g., ROS generation, ROS-dependent caspase activation, and reduction in mitochondrial membrane potential). These results support the use of iPSC-derived neurons to evaluate the neurotoxicity of ketamine.

### Mitochondrial dysfunction as a mechanism of ketamine toxicity

In the current study, a 100-μM dose of ketamine did not activate apoptosis, however it reduced ATP production and monoamine neurotransmitter reuptake activity in iPSC-derived neurons. Interestingly, the NADH/NAD^+^ ratio—a measure of oxidative stress—was actually elevated by the 100-μM dose of ketamine. Several previous studies have reported that volatile anesthetics (diethyl ether, halothane, enflurane, isoflurane) and pentobarbital increased NADH concentration in a rat myocardial model [26, 27]. Halothane has been reported to inhibit complex I activity and subsequently inhibit the mitochondrial electron transport system [28]. Barbiturates [29, 30] and midazolam [31] have also been reported to inhibit complex I activity. Therefore, we hypothesized that ketamine would cause complex I dysfunction and impair NADH utilization. In the biochemical assay for evaluating the direct effect of ketamine on bovine heart mitochondrial respiratory chain complexes, we observed that the activity of complexes I and V significantly decreased at the 125 and 500 μM doses of ketamine, whereas complexes II and IV were not affected. Hroudová and Fišar have reported that ketamine inhibited complex I in pig cerebral cortical mitochondria (IC50 = 361.6 ± 21.5 μM) [32], and Venâncio and colleagues have demonstrated that low-dose ketamine administration impaired complex I in an adult rat liver [33] and brain model [34]. Our data are consistent with these previous studies, as an increased NADH/NAD^+^ ratio was found in ketamine-treated iPSC-derived neurons. This could be explained by the impaired utilization of NADH caused by complex I inhibition. Furthermore, because mitochondrial oxidative phosphorylation is the major source of ATP production, complex I inhibition by the sub-apoptotic (100 μM) dose of ketamine may result in the progressive decrease in ATP production.

Interestingly, transmission electron microscopy analysis showed mitochondrial fragmentation and autophagosomes in the iPSC-derived neurons treated with 100 μM ketamine. Moreover, the confocal microscopy using fluorescent dye for activated mitochondria showed that 100 μM ketamine caused mitochondrial fission in neurons. These results suggest that mitochondrial dysfunction could be caused by a sub-apoptotic dose of ketamine, which is consistent with our results from the quantification of ATP production and NADH/NAD^+^ ratio. Mitochondria alter their shape (fusion or fission) depending on the cellular environment [35–37]. Changes in mitochondrial morphology have been linked to apoptotic cell death [38], and excessive fragmentation is associated with several chronic and acute neuropathological conditions [39]. In a stressful environment, mitochondria split into smaller pieces, and intracellular ROS production is accelerated. Previous studies on non-neuronal cells have suggested that changes in mitochondrial morphology may be essential for selecting damaged depolarized mitochondria for removal by autophagosomes (mitophagy) [40, 41]. Autophagy eliminates old and damaged mitochondria [42, 43], and maintains a healthy mitochondrial network. In this context, while 100 μM ketamine-induced toxicity may be overcome by autophagy related mechanisms, high-dose ketamine (500 μM) caused mitochondrial fission and degradation, which resulted in the loss of mitochondrial membrane potential and intracellular ROS generation. As a consequence, these changes induced the activation of caspases, and neuronal apoptosis. Further study is needed to reveal the relationship between ketamine-induced mitochondrial dysfunction and autophagy in human neurons.

Our study had some limitations. First, our data were obtained from cultured neurons. Because brain tissue consists of a complex network of neurons and glial cells, cell types other than dopaminergic neurons may affect the sensitivity to ketamine. Second, the iPSC-derived neural progenitors used in our experiments were derived from a single iPSC line. We cannot exclude the possibility of potential experimental variation between iPSC lines; however, we observed similar neurotoxic effects of ketamine in ReNcell experiments (Supplemental contents). In this context, the ketamine toxicity observed in our current study may not be limited to the hiPSC-derived cell line used here. Furthermore, the reproducibility of the results of the experiments using this hiPSC cell line is advantageous as an experimental model to test drug toxicity. Third, we observed neurotoxicity of ketamine at 100 μM and higher concentrations, which is a range higher than that used in clinical practice. However, in the clinical setting, brain tissue can be influenced by several aggravating factors, such as concomitant use of several anesthetics [44], hypoxia and surgery-induced inflammation. In these situations, ketamine may cause neurotoxicity at lower concentrations. Fourth, we did not investigate whether NMDA receptor is associated with ketamine neurotoxicity in hiPSC-derived neurons. It has been reported that ketamine upregulated NMDA receptors, which was followed by toxic influx of calcium into neurons, leading to membrane potential depolarization, elevated ROS generation and caspase activation [44–46]. In constrast, other studies have indicated that ketamine-induced mitochondrial dysfunction may be mediated by NMDA-independent pathways in hepatocytes and human lymphocytes [20, 47]. Further studies are needed to reveal the relationship between neurotoxicity and NMDA receptor in human neurons. Finally, the stage of neural development equivalent to the cultured human iPSC-derived dopaminergic neurons is unknown. Further studies are required to relate our findings to the stage of neural development.

In conclusion, we established an *in vitro* model for assessing the neurotoxicity of ketamine to human iPSC-derived neurons. Ketamine at 100 μM reduced ATP production, increased the NADH/NAD^+^ ratio and promoted mitochondrial fission. Ketamine directly inhibited the activity of mitochondrial respiratory complexes I and V, which could affect NADH utilization and ATP levels in neurons. At higher concentrations, ketamine caused mitochondrial degradation and ROS generation, which can activate apoptosis. These results suggest that initial mitochondrial dysfunction underlies ketamine-induced neural toxicity. Thus, it is important that the plasma concentrations of ketamine be tightly controlled to avoid the deleterious neurotoxic effects of ketamine on neurons.

## Supporting Information

S1 Fig Quantitative analysis of ketamine toxicity in the cortical neuronal cell line. (PDF)

S2 Fig Oxidized and reduced forms of NAD in the cortical neuronal cell line after ketamine treatment. (PDF)

S3 Fig Representative transmission electron microscopy images of cortical neuronal cell line treated with ketamine. (PDF)
