# Supplementary material for: Ketamine Causes Mitochondrial Dysfunction in Human Induced Pluripotent Stem Cell-Derived Neurons
Source: PLoS One. 2015 May 28;10(5):e0128445. doi: 10.1371/journal.pone.0128445 (PMC4447382; doi:10.1371/journal.pone.0128445)
Supplement: S1 Fig — (PDF) [file pone.0128445.s001.pdf]

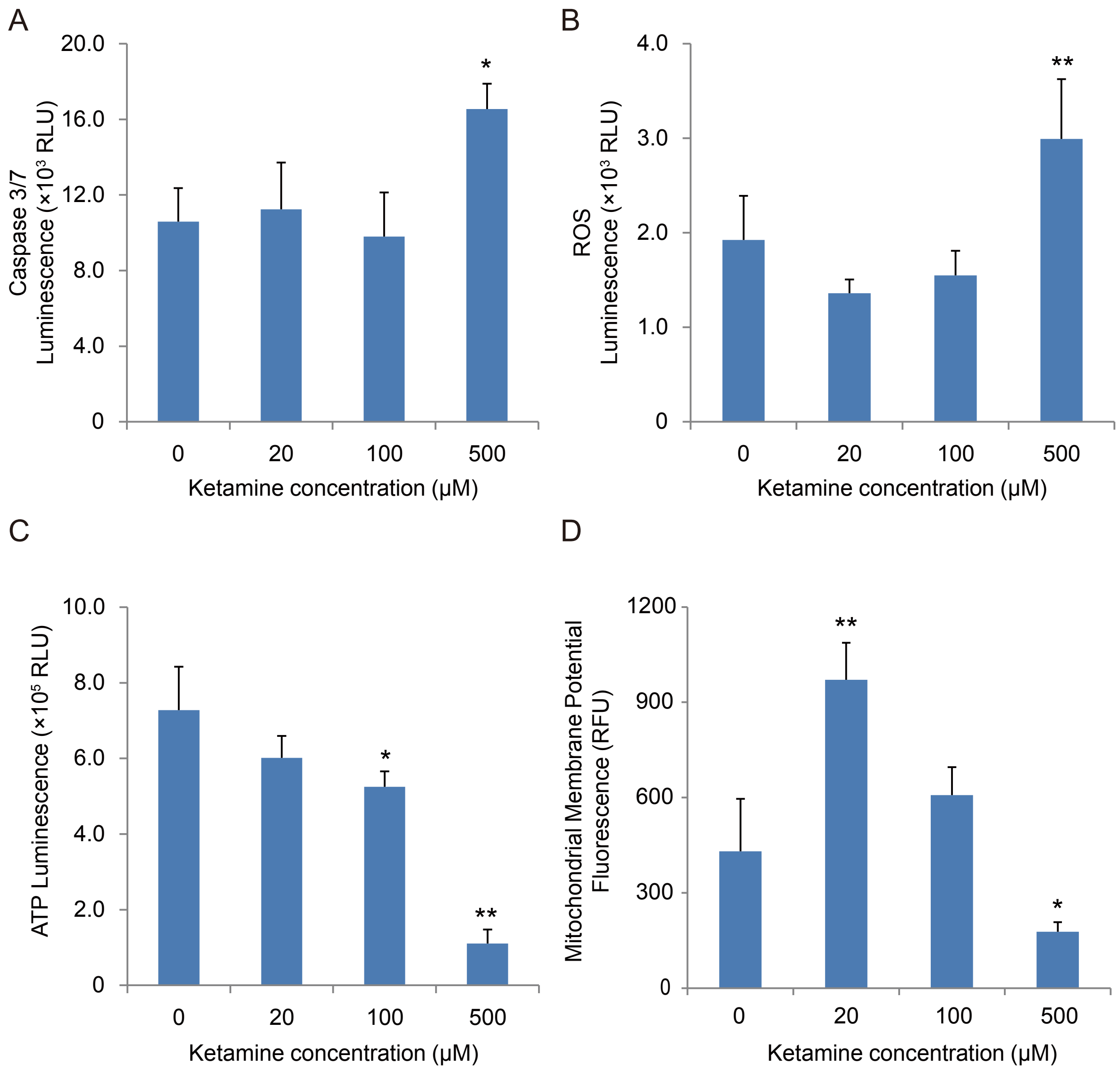

Fig. S1. Quantitative analysis of ketamine toxicity in a cell line derived from human fetal cortical brain tissue. (A) Caspase 3/7 activity after the exposure to ketamine (20, 100, and 500 μM) for 24 h. Caspase 3/7 activity was significantly increased with the 500 μM dose of ketamine ($1.7 \times 10^4 \pm 1.3 \times 10^3$ RLU in 500 μM vs. $1.1 \times 10^4 \pm 1.8 \times 10^3$ in 0 μM, $P = 0.02$). (B) Reactive oxygen species (ROS) in the cortical neuronal cell line after treatment with ketamine (20, 100, and 500 μM) for 24 h. ROS generation was significantly increased following treatment with 500 μM ketamine ($3.0 \times 10^4 \pm 6.3 \times 10^3$ RLU in 500 μM vs. $1.9 \times 10^4 \pm 4.7 \times 10^3$ in 0 μM, $P = 0.009$). (C) Cellular ATP production in cortical neuronal cell line.

treated with ketamine were compared with untreated control cells ($7.3 \times 10^5 \pm 1.2 \times 10^5$ RLU). ATP production was significantly decreased by 100 μM ketamine ($5.3 \times 10^5 \pm 4.1 \times 10^4$ RLU, $P = 0.02$), and by 500 μM ($1.1 \times 10^5 \pm 3.7 \times 10^4$ RLU, $P = 0.00002$) in a dose-dependent manner. (D) Mitochondrial membrane potential in the ketamine-treated cortical neuronal cell line. The highest concentration of ketamine (500 μM) significantly reduced mitochondrial membrane potential level ($1.8 \times 10^2 \pm 0.30 \times 10^2$ RFU in 500 μM vs. $4.3 \times 10^2 \pm 1.6 \times 10^2$ in 0 μM, $P = 0.02$). Carbonyl cyanide 3-chlorophenylhydrazone, which disrupts the mitochondrial membrane potential, was used as a positive control. All data were extracted from the fluorescence of 4 μM carbonyl cyanide 3-chlorophenylhydrazone-treated neurons. Data are presented as means ± SD; n = 4 in each experiment. * $P < 0.05$, ** $P < 0.01$, respectively, compared with 0 μM. RLU = relative light units; RFU = relative fluorescence units.
