# Supplementary material for: Ketamine Causes Mitochondrial Dysfunction in Human Induced Pluripotent Stem Cell-Derived Neurons
Source: PLoS One. 2015 May 28;10(5):e0128445. doi: 10.1371/journal.pone.0128445 (PMC4447382; doi:10.1371/journal.pone.0128445)
Supplement: S2 Fig — (PDF) [file pone.0128445.s002.pdf]

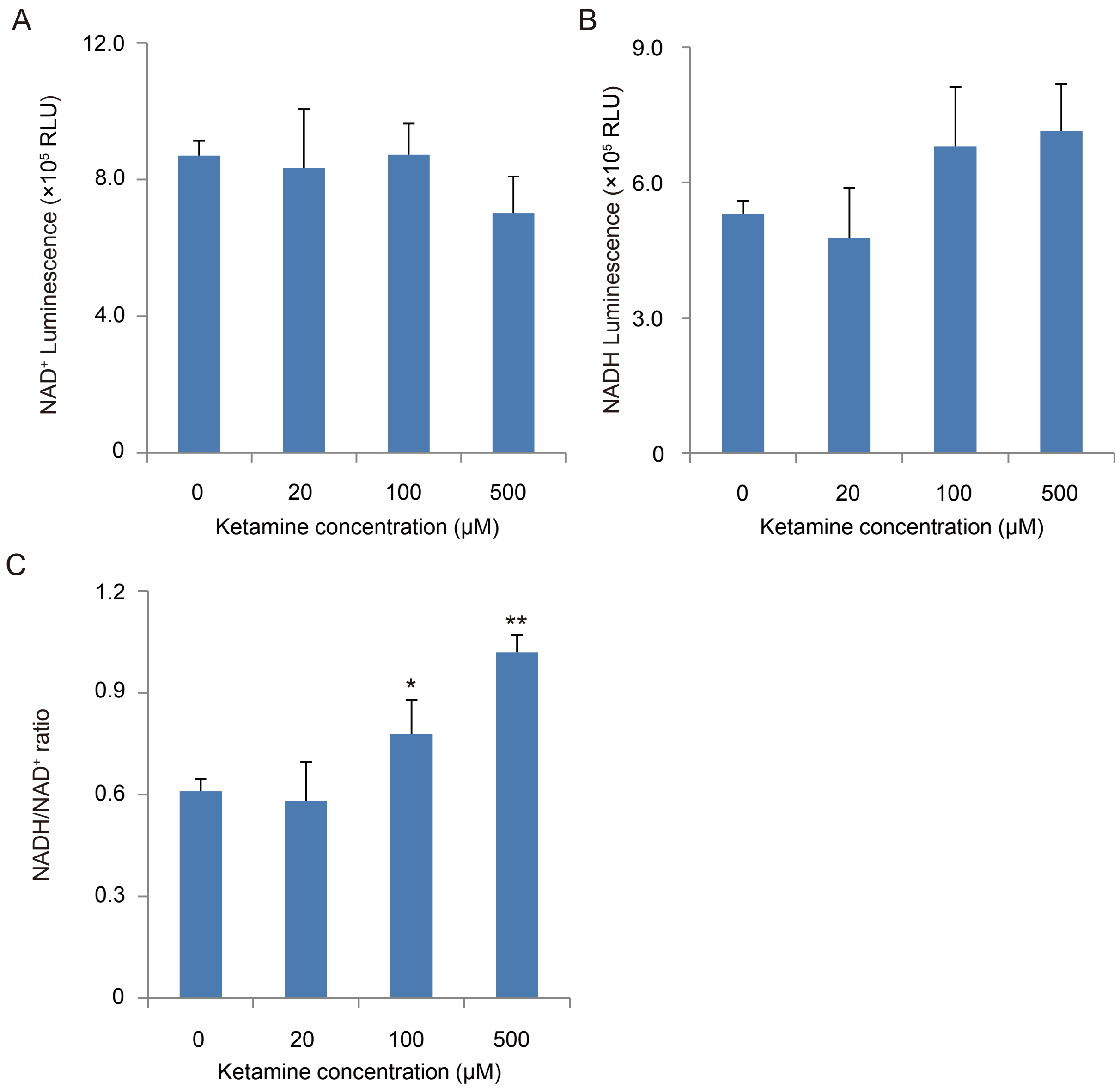

S2 Fig. Oxidized and reduced forms of NAD in the cortical neuronal cell line after ketamine treatment. (A) NAD^+, (B) NADH, and (C) $NADH/NAD^+$ ratio after treatment with ketamine (20, 100, and 500 μM) for 24 h. Compared with the control (0 μM) (0.61 ± 0.036), the $NADH/NAD^+$ ratio was significantly higher in the cells treated with 100 μM (0.78 ± 0.10, $P = 0.03$) and 500 μM (1.02 ± 0.051, $P = 0.00008$) ketamine. Results are presented as means ± SD; n = 4 in each experiment. * $P < 0.05$, ** $P < 0.01$ respectively, compared with 0 μM. RLU = relative light unit.
