# Supplementary material for: Ketamine Causes Mitochondrial Dysfunction in Human Induced Pluripotent Stem Cell-Derived Neurons
Source: PLoS One. 2015 May 28;10(5):e0128445. doi: 10.1371/journal.pone.0128445 (PMC4447382; doi:10.1371/journal.pone.0128445)
Supplement: S3 Fig — (PDF) [file pone.0128445.s003.pdf]

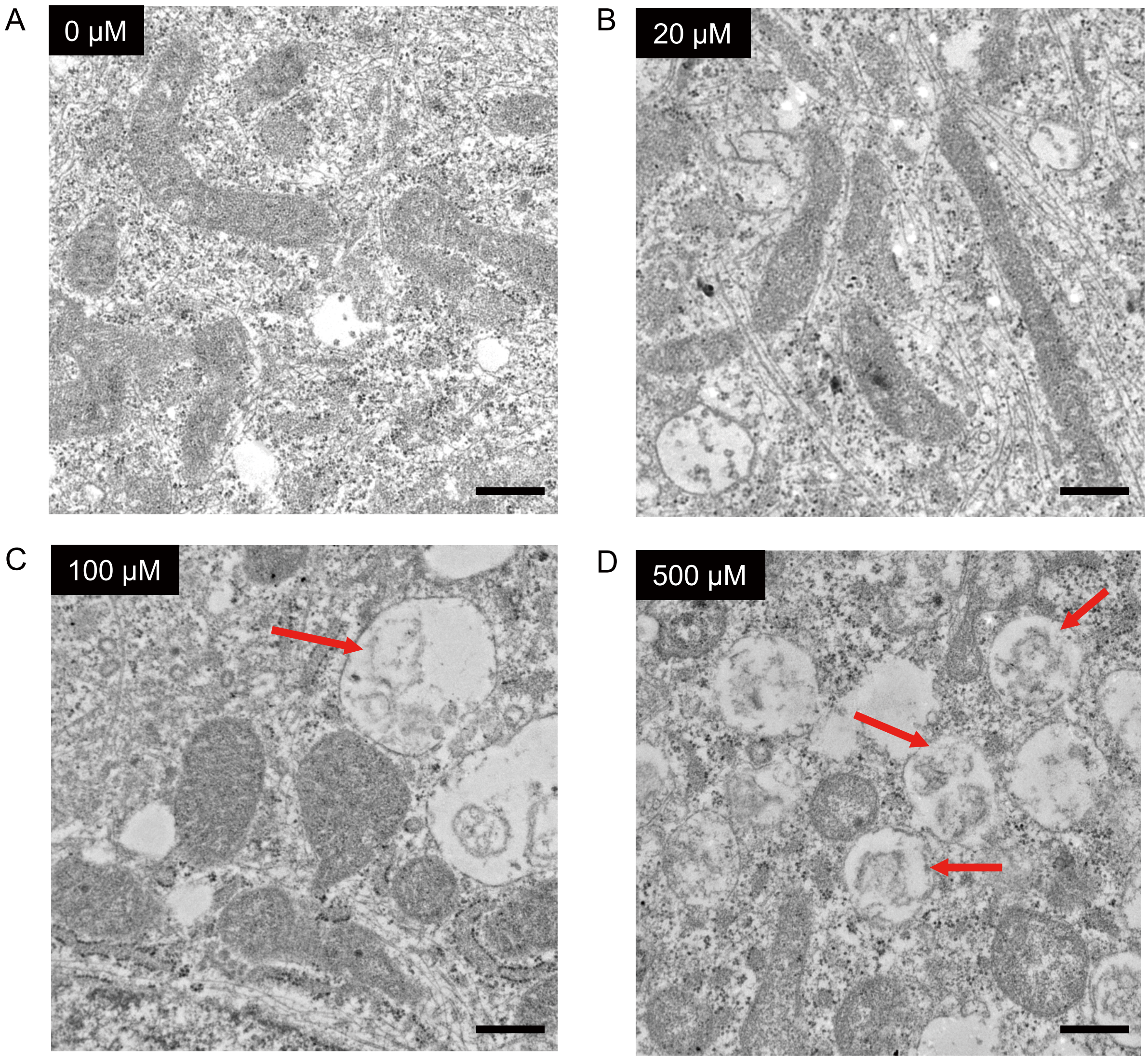

S3 Fig. Representative transmission electron microscopy images of cortical neuronal cell line treated with ketamine. Control neurons (A) and 20 μM ketamine-treated neurons (B) had an abundance of elongated mitochondria with intact inner and outer membranes. (C) In the cells treated with 100 μM of ketamine, the number of elongated mitochondria decreased, and there was indication of mitochondrial fragmentation. Autophagosomes (arrow) were observed in some neurons. (D) Cells treated with 500 μM ketamine. The lengths of mitochondria were short and the number of autophagosomes increased. *Scale bar* = 500 nm.
